# Bacterial competition and quorum‐sensing signalling shape the eco‐evolutionary outcomes of model *in vitro* phage therapy

**DOI:** 10.1111/eva.12435

**Published:** 2016-12-20

**Authors:** Rachel Mumford, Ville‐Petri Friman

**Affiliations:** ^1^ Silwood Park CampusImperial College LondonAscotBerkshireUK; ^2^ Department of BiologyUniversity of YorkYorkUK

**Keywords:** coevolution, competition, cost of resistance, host–parasite interactions, phage therapy, polymicrobial infections, quorum‐sensing signalling, resistance

## Abstract

The rapid rise of antibiotic resistance has renewed interest in phage therapy – the use of bacteria‐specific viruses (phages) to treat bacterial infections. Even though phages are often pathogen‐specific, little is known about the efficiency and eco‐evolutionary outcomes of phage therapy in polymicrobial infections. We studied this experimentally by exposing both quorum‐sensing (QS) signalling PAO1 and QS‐deficient *lasR Pseudomonas aeruginosa* genotypes (differing in their ability to signal intraspecifically) to lytic PT7 phage in the presence and absence of two bacterial competitors: *Staphylococcus aureus* and *Stenotrophomonas maltophilia*–two bacteria commonly associated with *P. aeruginosa* in polymicrobial cystic fibrosis lung infections. Both the *P. aeruginosa* genotype and the presence of competitors had profound effects on bacteria and phage densities and bacterial resistance evolution. In general, competition reduced the *P. aeruginosa* frequencies leading to a lower rate of resistance evolution. This effect was clearer with QS signalling PAO1 strain due to lower bacteria and phage densities and relatively larger pleiotropic growth cost imposed by both phages and competitors. Unexpectedly, phage selection decreased the total bacterial densities in the QS‐deficient *lasR* pathogen communities, while an increase was observed in the QS signalling PAO1 pathogen communities. Together these results suggest that bacterial competition can shape the eco‐evolutionary outcomes of phage therapy.

## 1 Introduction

Growing concern for the evolution of antibiotic resistant bacteria and in particular for multiresistant gram‐negative bacteria (Levy & Marshall, 2004), has led to renewed interest in alternative treatments including phage therapy (Rossolini, Arena, Pecile, & Pollini, 2014). Phage therapy—the use of pathogen‐specific parasitic viruses (bacteriophages) as a treatment for bacterial infections—is almost hundred years old and has been used for decades to treat bacterial infections in Eastern European countries such as Georgia and Poland (Abedon, Kuhl, Blasdel, & Kutter, 2011; Alisky, Iczkowski, Rapoport, & Troitsky, 1998; Housby & Mann, 2009). While many studies have demonstrated the safety and benefits of phage therapy (Abedon et al., 2011; Merabishvili et al., 2009; Rose et al., 2014), phages have not yet been incorporated into western medicine partly due to lack of proper clinical trials and historically inconsistent treatment results (Kutateladze & Adamia, 2008). While large‐scale clinical trials are currently under way (e.g. Phagoburn; (Expert round table on, acceptance and therapy re‐implementation of bacteriophage, 2016)), the evolutionary outcomes of phage therapy are relatively unknown. Recent studies have shown that bacteria and phages can rapidly coevolve during model phage therapy treatments (Betts, Vasse, Kaltz, & Hochberg, 2013; Friman et al., 2016) and that the diversity of phage communities can affect the bacterial resistance evolution (Betts, Gifford, MacLean, & King, 2016; Hall, De Vos, Friman, Pirnay, & Buckling, 2012). Besides rapid coevolution, further complications could arise from interspecific bacterial competition due to polymicrobial nature of bacterial infections: many human infections contain multiple different pathogenic bacterial and other microbial species (Peters, Jabra‐Rizk, O'May, Costerton, & Shirtliff, 2012). Considerable genotypic variation also exists between different strains of a pathogen, and this variation is known to differ between different patients and to affect the pathogen susceptibility to phages (Debarbieux et al., 2010; Essoh et al., 2013; Friman, Ghoul, Molin, Johansen, & Buckling, 2013). Understanding the relative importance and interactive effects of these potentially complicating factors is thus crucial for developing reliable and consistent phage therapy treatments. Here, we focused explicitly on the ecological and evolutionary outcomes of phage therapy in polymicrobial communities and asked how focal bacterial genotype and the competition with other bacterial pathogens affect the total bacterial loads and focal pathogen resistance evolution during *in vitro* model phage therapy.

The bacterium *Pseudomonas aeruginosa* is an opportunistic pathogen that commonly infects many immunocompromised patients including cystic fibrosis (CF) and burn victim patients (Harrison, 2007; Turner, Everett, Trivedi, Rumbaugh, & Whiteley, 2014). *P. aeruginosa* is often characterized by multidrug resistance to conventional antibiotics (Strateva & Yordanov, 2009), and hence, the development of novel phage therapy treatments could potentially help a large number of patients (Harper & Enright, 2011). While *P. aeruginosa* can rapidly evolve resistance to various bacteriophages, which could decrease the feasibility and long‐term benefits of phage therapy (Betts et al., 2013; Friman et al., 2013; Hall et al., 2012), it has also been shown that phages can counteract resistance evolution by coevolving to be more infective (Betts et al., 2016; Friman et al., 2016). However, it is less clear how important these coevolutionary dynamics are in more complex microbial communities. For example, lung and wound infections are often very diverse and consist of multiple different nonpathogenic and pathogenic bacterial species (Folkesson et al., 2012; Harrison, 2007; Korgaonkar, Trivedi, Rumbaugh, & Whiteley, 2013) that could modify phage effects indirectly via competition.

Competition could affect the evolution of phage resistance via demographic and genetic effects. Firstly, competition is likely to reduce focal pathogen population densities which could weaken the selection for resistance due to less frequent phage–bacteria encounter rates and lowered supply of resistance mutations (Levin & Bull, 2004; Lopez‐Pascua & Buckling, 2008). These demographic effects could be occurring indirectly via competition for shared resources in the site of infection or directly via interference competition via bacteria‐specific toxins such as bacteriocins (Ghoul et al., 2015; Inglis, Gardner, Cornelis, & Buckling, 2009). Furthermore, *P. aeruginosa* has been shown to display greater virulence, antibiotic tolerance and growth when cocultured with gram‐positive *S. aureus* bacterium (Korgaonkar et al., 2013; Michelsen et al., 2014), which suggests that the presence of other bacterial species could also facilitate target pathogen coexistence in polymicrobial infections. Secondly, there might be trade‐offs between evolving phage resistance and retaining competitive ability or virulence due to conflicting selection pressures (Friman & Buckling, 2014). Such trade‐offs are often manifested as antagonistic pleiotropy where a mutation in the gene that confers benefit in the presence of phage has a negative effect on some other function such as uptake of nutrients (Lenski & Levin, 1985). The magnitude of such trade‐offs is often dependent on environmental conditions, being larger in nutrient‐poor environments (Yoshida, Hairston, & Ellner, 2004) or in the presence of competitors (Kassen, 2002). Lastly, it has been shown that the presence of a phage can change the competitive interactions between different bacterial species and that this effect depends on which competing bacterial species is affected by the phage (Harcombe & Bull, 2005).

The effect of competitors on focal pathogen fitness, and pathogen potential to evolve resistance to phages, could further depend on the focal pathogen genotype. For example, *P. aeruginosa* CF lung infections are genetically diverse and this heterogeneity is driven by both temporal (Marvig, Madsen, Molin, & Johansen, 2014) and spatial variations (Jorth et al., 2015). It has been recently shown that phages can have a different effect on *P. aeruginosa* density and resistance evolution depending on the strain and the genotype; specifically, the time bacteria spent adapting to the lung environment seems to make bacteria more susceptible to phages (Friman et al., 2013, 2016). One notable adaptation to the CF lung environment is the loss of quorum‐sensing related traits (Andersen, Marvig, Molin, Krogh Johansen, & Griffin, 2015; Marvig et al., 2014; Michelsen et al., 2014). Quorum sensing (QS) is a means by which bacteria communicate through the release of signalling molecules allowing cells to carry out density‐dependent gene expression (Miller & Bassler, 2001). In *P. aeruginosa,* the ability to quorum sense is critical for controlling behaviours such as the production of virulence factors (Folkesson et al., 2012) and it is known that strains from acute infections (early colonisations) are more virulent compared to strains from chronic infections (long‐term colonisations) (Marvig et al., 2014; Smith et al., 2006). Interestingly, recent evidence suggests that QS‐regulated genes can also affect bacterial resistance to phages. For example, with *E. coli,* QS genes regulate resistance to phage plastically via reduction of cell surface receptors (Hoyland‐Kroghsbo, Maerkedahl, & Svenningsen, 2013; Taj, Samreen, Hassani, Taj, & Yunlin, 2014). Similarly, QS has been shown to be an important “switch” for choosing between different antiphage defence strategies in the bacterium *Vibrio anguillarum* (Tan, Svenningsen, & Middelboe, 2015). As a result, the decrease of phage resistance in *P. aeruginosa* QS mutants could be due to the loss of functional QS genes.

Here, we used in vitro experimental evolution approach to study the eco‐evolutionary outcomes of phage therapy with *P. aeruginosa* focal pathogen that frequently co‐infects the lungs of patients with CF (Harrison, 2007). We manipulated both the presence of *Staphylococcus aureus* and *Stenotrophomonas maltophilia* competitors (one or two competitors present–our definition of a polymicrobial community from here on) and the PT7 phage and used two *P. aeruginosa* pathogen genotypes: QS signalling PAO1 strain and QS‐deficient *lasR* mutant strain, which does not produce or respond to QS signals (Diggle, Griffin, Campbell, & West, 2007). These bacterial species were chosen because they commonly coexist and infect human patients suffering from burn wounds or cystic fibrosis (CF) lung infections (Harrison, 2007). We used fully factorial design where both *P. aeruginosa* genotypes were evolved in all possible combinations and measured bacterial and phage densities and coevolutionary changes between *P. aeruginosa* and PT7 phage at the end of the selection experiment. We hypothesized that the rate of phage resistance evolution could be negatively affected by competition via negative effects on population densities (lowered mutation supply rate and phage–bacteria encounter rates) and that the effect of competition could further depend on the focal pathogen genotype, the composition of competitor community and the pleiotropic costs of adaptation.

## 2 Materials and Methods

### 2.1 Bacterial and phage strains

In addition to *P. aeruginosa* (Diggle et al., 2007)*,* we used *Staphylococcus aureus subsp. aureus* (DSM‐20231) and *Stenotrophomonas maltophilia* (DSM‐50170) bacteria in our experiments. We chose *P. aeruginosa* as our focal species as it is one of the most common causes of morbidity for patients with CF, while *S. aureus* and *S. maltophilia* often coexist with *P. aeruginosa* among *Haemophilus influenza, Streptococcus pneumoniae, Burkholderia cenocepacia, Ralstonia and Achromobacter* (Folkesson et al., 2012; Jelsbak et al., 2007). To compare the effect of pathogen genotype, two strains of *P. aeruginosa* were used: QS signalling PAO1 and QS‐deficient PAO1 *lasR* mutant strains (Diggle et al., 2007). Apart from the mutation in QS signalling pathway, the two isolates were otherwise isogenic (Fletcher et al., 2007). The *lasR* mutation is often associated with isolates from the later stages of long‐term infections in patients with CF (Andersen et al., 2015; Marvig et al., 2014), and its weakened virulence is due to inability to detect and produce quorum‐sensing signalling molecules that activate the expression of *P. aeruginosa* virulence factors (Smith et al., 2006). A lytic bacteriophage, PT7, which obligately kills *P. aeruginosa*, was used as a phage (Friman et al., 2016). Relatively little is known about PT7 phage. Even though its genome has not been sequenced, previous studies suggest that it is not closely related to PB1‐like or phiKMV‐like phages (Merabishvili et al., 2007). Similarly, it is unclear which receptors it uses to infect *P. aruginosa*. Prior the experiment, we confirmed that phage PT7 was not able to infect *S. aureus* or *S. maltophilia* (tested with streak assays) and that the presence of *S. aureus* or *S. maltophilia* had no effect on phage densities during short‐term cocultivation (24 hr). Moreover, both the PAO1 and *lasR* strains were susceptible to phage PT7 in the beginning of the experiment (streak assays) yielding similar phage population densities (phage efficiency of plating with plaque essay: ~10^8^ phage particles ml^−1^ from the same ancestral phage stock).

### 2.2 Experimental design, growth conditions and selection experiment

We used a factorial design to independently manipulate bacterial community composition, the presence of phage and *P. aeruginosa* genotype. To this end, *P. aeruginosa* focal pathogen strains, PAO1 and *lasR*, were evolved in both the absence and presence of phage under four different competition treatments: alone, with *S. aureus,* with *S. maltophilia* and with both *S. aureus* and *S. maltophilia*. Each treatment (16 in total) was replicated five times.

The communities were grown in 1.5 ml of 10% nutrient broth (NB) media (containing 0.5 g peptone and 0.3 g beef extract per litre distilled water) in deep 96‐well plates (Starlab; 2.2 ml of total volume). All treatments were inoculated with approximately 3.8 × 10^5^ bacterial cells per ml, where two‐competitor treatments were inoculated with 1:1 ratio of both bacteria and three‐competitor treatments with 1:1:1 ratio of every bacteria. Approximately 1.5 × 10^8^ phage particles were added to all phage treatments. All populations were incubated as static cultures at 37°C to reflect human body temperature. The selection experiment was run for 16 days with transfers carried out every fourth day. At each transfer, the cultures were first mixed and homogenized using a pipette before an inoculum of 250 μl was transferred to new deep‐well plates containing 1.5 ml fresh media in each well, after 500 μl of each microbial community was cryopreserved in 20% of glycerol at −80°C. Given nutrient broth concentration was chosen to allow prolonged growth during 4‐day transfer intervals and to reduce the *P. aeruginosa* biofilm and exopolymer production.

### 2.3 Bacterial and phage density measurements

Bacterial densities were measured only at the end of the experiment by serially diluting the samples isolated from the last time point and plating out 10 μl of each dilution onto NB agar plates (100% NB media supplemented with 12 g agar per litre). To determine *P. aeruginosa* densities in multispecies communities, community treatment samples were also plated on *Pseudomonas* selective agar plates (16 g Peptic digest of animal tissue, 10 g Casein enzymic hydrolysate, 10 g K_2_SO_4_, 1.4 g MgCl_2_·6H_2_O, 10 ml glycerol and 11 g Agar per litre with 200 mg C‐N selective supplement dissolved in 4 ml 1:1 ethanol:distilled water). Bacteria were incubated at 37°C for 48 hr before counting the colonies and calculating the number of colony‐forming units (CFU) per ml. At every transfer, phages were extracted by mixing with 10% chloroform to kill the bacteria. After vortexing and centrifugation, chloroform‐free phage supernatants were stored at 4°C. Phage densities were estimated at every transfer with plaque assays where phage densities are defined as growth on a lawn of ancestral PAO1 bacterial strain. PAO1 ancestral strain was grown at 37°C for 24 hr, and 200 μl of this culture was then mixed with 20 ml of 50°C soft agar and poured in an even overlay over square NB agar plates. A 10 μl of phage serial dilutions (10^−4^–10^−7^) was then pipetted onto the surface of the pseudomonas–agar overlay, plates were incubated at 37°C for 24 hr and the number of phage plaques, that is phage particles, counted.

### 2.4 Phage resistance assays

A streak assay methodology was used to estimate the evolution of bacterial resistance and phage infectivity (Buckling & Rainey, 2002). A total of 12 randomly chosen colonies per each *P. aeruginosa* population were isolated at the end of the experiment and grown in 96‐well microplates at 37°C in 150 μl of NB media. After 24‐hour growth, colonies were cryopreserved at −80°C as above for evolutionary analyses. Phage resistance was measured by pipetting 25 μl of phage in a line across square NB plates. A sterilised 12‐pin replicator (V&P Scientific) was then used to streak 12 bacterial colonies across the dried line of phage. Plates were incubated at 37°C for 24 hr (or until the bacterial streak became visible). Colonies with a clear reduction in growth over the phage line were scored as susceptible (0) and with normal growth over the phage as resistant (1). Phage resistance was determined at the population level in terms of a proportion of resistant colonies per population. All *P. aeruginosa* colonies were tested against the ancestral PT7 phage and evolved PT7 phages isolated from their own population (coevolved phage population isolated by the way of chloroforming as described above).

### 2.5 Measuring the pleiotropic cost of adaptation

The pleiotropic cost of adaptation was measured as the final bacterial density at 48 hr using the same colonies that were used in the phage resistance assays. Colonies were inoculated in 96‐well microplates containing 200 μl NB media per well using a sterilised 96‐pin replicator (Boenik). The plates were then grown at 37°C and optical density (OD_600_) measurements taken after 48 hr. The growth of the colonies, which had been subjected to competition and/or phages in the selection experiment, was compared to colonies that had evolved alone. A mean population density was calculated for all the colonies isolated from the same population. Even though this method results in indirect fitness measures, it was the only practical way to estimate the cost due to a high number of evolved colonies (960 clones).

### 2.6 Statistical analysis

All models and test statistics are presented in the Tables S1–S5. For the bacterial density data, a linear model was fitted predicting square root‐transformed *P. aeruginosa* density as a function of phage treatment, competition and pathogen genotype. For the phage density data, a mixed model was used for log‐transformed phage density data as a function of competition and pathogen genotype with time set as a repeated factor. For the phage resistance data, a linear model was fitted predicting arcsine‐transformed resistance data as a function of phage evolution (ancestral or coevolved), pathogen genotype and competition. A similar model was used for data predicting the cost of adaptation with the exception that untransformed bacterial growth data were used for the analysis. Post hoc tukey honest significance difference tests were carried out to further investigate significant interactions between factor levels. All analyses were conducted in R, version 3.1.2. (R Core Team. 2014).

## 3 Results

### 3.1 Bacterial and phage densities during the selection experiment

**Figure 1:**
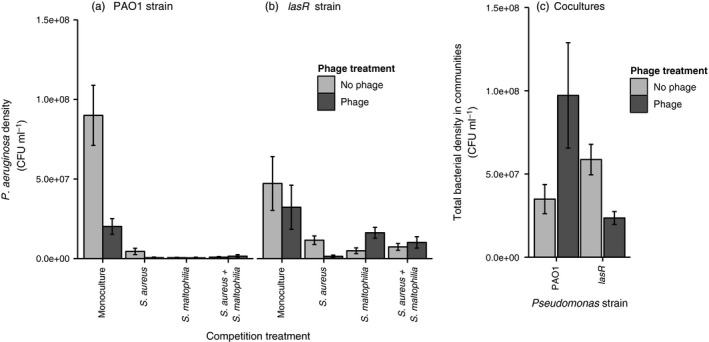
The comparison of *Pseudomonas aeruginosa* (panels a and b) and total bacterial population densities (panel c) at the end of the selection experiment between different treatments (CFU denotes for colony‐forming units per ml). Panel c shows the mean over all competition treatments for PAO1 and *lasR* strains, respectively. All bars show ±1 *SEM*

Both phages (*F*_1, 64_ = 8.67, *p* = .005) and competitors (*F*_3, 64 _= 48.80, *p* < .001) significantly reduced *P. aeruginosa* densities at the end of the selection experiment (Figure 1a,b, Table S1). In the absence of phages, both PAO1 and *lasR* monocultures had higher *P. aeruginosa* densities compared to all polymicrobial communities, and PAO1 strain reached higher population densities compared to *lasR* strain when evolving in the absence of a phage and competitors (*p* < .001 for all comparisons). However, the relative effect of competition was stronger for the PAO1 strain (genotype × competition: *F*_3, 64_ = 5.02, *p* = .003). Moreover, phages reduced the densities of PAO1 strain more compared to a *lasR* strain (phage × competition: *F*_3, 64_ = 7.70, *p* < .001). The phage effect depended also on the type of competitive community: in general, phage had a negative effect on *P. aeruginosa* in the presence of *S. aureus* regardless of the pathogen genotype, while phages had mainly nonsignificant effects in the other polymicrobial communities (and even a positive effect in the presence of *S. maltophilia*, Figure 1a,b). Unexpectedly, phage selection also affected the total bacterial biomasses in the polymicrobial communities (Figure 1c, Table S2) by increasing the total bacterial densities in the PAO1 communities, and decreasing the total bacterial densities in the *lasR* communities in general (genotype × phage: F_1, 56_ = 8.04, *p* = .006; the effect varied depending on the community composition, Figure S1).

**Figure 2:**
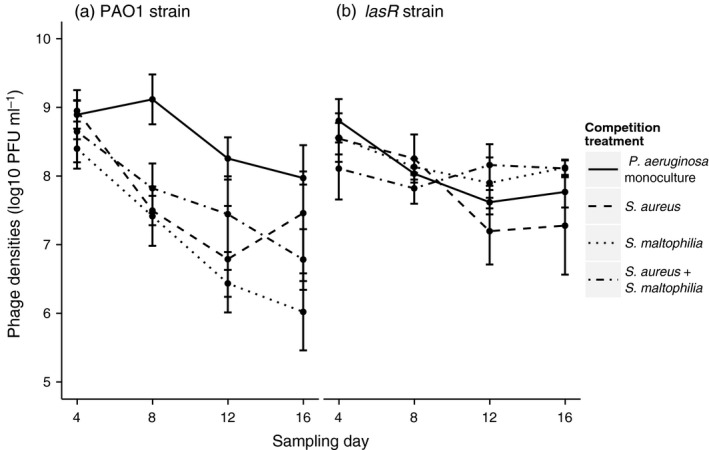
Phage population densities in PAO1 (panel a) and *lasR* (panel b) focal pathogen communities in the absence and presence of competitors (PFU denotes for plaque‐forming units, that is phage particles per ml). All bars show ±1 *SEM*

Phage densities decreased during the selection experiment in general (Time: *F*_3, 30.35_ = 17.34, *p* < .001, Figure 2a,b, Table S3). While competition had no significant main effect on the phage densities, a significant interaction was found: even though competition had no effect in the weakly virulent pathogen communities, it reduced the phage densities in the PAO1 pathogen communities (genotype × competition: *F*_1, 32.1_ = 2.96, *p* = .047, Figure 2a,b). The number or type of competitors did not affect the phage densities with either PAO1 or *lasR* strain (*p* > .05 in all comparisons). Together these results suggest that competitors had stronger negative effects on both the bacteria and phages in the PAO1 compared to *lasR* pathogen communities.

### 3.2 Bacteria–phage coevolution in different communities

**Figure 3:**
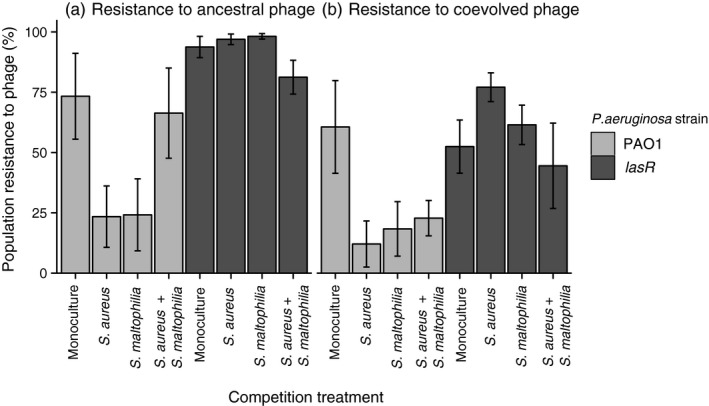
The resistance of evolved PAO1 (light grey) and *lasR* (dark grey) strains to ancestral and coevolved phages measured at the end of the experiment. Competition treatment shows the absence and presence of competitors during the selection experiment. Only populations that had evolved in the presence of phage were used for the analysis; all *P. aeruginosa* populations that had evolved in the absence of phage were susceptible to phages. All bars show ±1 *SEM*

Both initially susceptible PAO1 and *lasR* strains evolved increased the levels of resistance to ancestral phage (Figure 3a,b, Table S4), while the *lasR* strain evolved higher levels of resistance compared to the PAO1 strain in general (genotype: *F*_1, 62_ = 35.94, *p* < .001). While competitors had no effect on the *lasR* strain resistance evolution, they generally constrained PAO1 resistance evolution (phage origin × competition: *F*_1, 62_ = 6.94, *p* < .001) with all competitive communities having similar effects (*p* > .05 in all comparisons). We also found that phages coevolved to become more infective during the selection experiment (Figure 3a,b): the resistance of evolved bacteria was lower when measured against evolved compared to ancestral phages (phage origin: *F*_1, 62_ = 25.38 *p* < .001). Interestingly, PAO1 resistance was less affected by phage coevolutionary history (ancestral vs coevolved) compared to *lasR* strain (phage origin × genotype: F_1, 62_ = 4.15, *p* = .046). Together these results suggest that competition altered the trajectory of bacteria–phage coevolution.

### 3.3 Pleiotropic cost of adaptation

**Figure 4:**
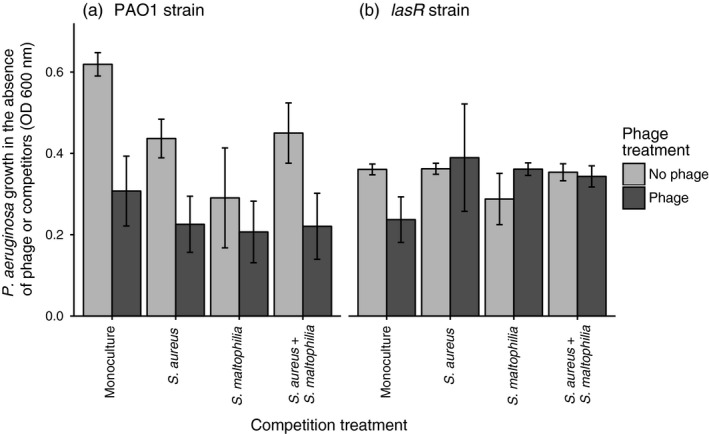
The cost of adaptation measured in terms of maximum population density after 48 hr of growth. Panel (a) shows the growth of evolved PAO1 and panel (b) the growth of evolved *lasR* strain in the absence of phage or competitors at the end of the selection experiment. Phage and competition treatments denote the absence and presence of a phage and competitors during the selection experiment. All bars show ±1 *SEM*

Coevolutionary history with the phage led to reduced bacterial growth in the absence of phages (*F*_1, 71_ = 13.36, *p* < .001, Figure 4a‐b, Table S5). While the focal pathogen genotype (*F*_1, 71_ = 2.34, *p* = .131) or the presence of competitors (*F*_1, 71_ = 1.88, *p* = .175) had nonsignificant main effects on the pathogen growth, the growth cost imposed by phage selection was larger with the PAO1 strain (genotype × phage: *F*_1, 71_ = 6.27, *p* = .015). Moreover, already the presence of competitors led to reduced PAO1 strain growth in the absence of phage selection (genotype × competition: *F*_1, 71_ = 7.08, *p* = .010; all competitive communities had similar effects: *F*_3, 63_ = 2.38, *p* = .078). Consistent with the population density data, the evolved PAO1 strain reached higher population densities compared to *lasR* strain when bacteria had evolved in the absence of a phage and competitors (genotype × phage: *F*_1, 71_ = 6.27, *p* = .015). These results suggest that even though both pathogen genotypes suffered from a reduced growth due to phage selection in monocultures, only the PAO1 strain was affected by the presence of competitors and hence suffered relatively higher pleiotropic cost of adaptation in polymicrobial communities.

## 4 Discussion

Here, we studied the role of bacterial competition for the efficiency and eco‐evolutionary outcomes of phage therapy in model polymicrobial infections in vitro. We found that both phages and competitors reduced the focal pathogen densities. However, this effect was strongly dependent on the focal pathogen genotype with both competitors and phages having a more severe effect on the QS signalling PAO1 strain. The negative effects of competition observed at the population level correlated with reduced rate of resistance evolution. Interestingly, phage presence decreased the total bacterial densities in *lasR* pathogen communities demonstrating an unexplored potential benefit of phage therapy: indirect, community‐wide reduction in pathogenic bacterial loads in polymicrobial infections. However, a converse pattern was observed in PAO1 communities, which suggest that phages could also indirectly worsen the polymicrobial infections by increasing the density of other pathogenic bacteria. Together these results suggest that phage‐mediated effects depend on bacterial competition and the focal pathogen genotype pinpointing the need to understand eco‐evolutionary consequences of phage therapy in the community context.

Both competitors and phages had a negative effect on *P. aeruginosa* densities while the effect of competition was relatively larger compared to the effect of a phage. While the number or the composition of competitive communities had no clear effects on *P. aeruginosa* densities, the effect of competition depended on the *P. aeruginosa* genotype being more severe for the PAO1 compared to *lasR* strain in general. This suggests that QS may play an important role for *P. aeruginosa* competition against other bacterial species. For example, the proportion of *lasR* mutants typically increases during chronic polymicrobial CF infections (Ghoul et al., 2015; Marvig et al., 2014; Smith et al., 2006) and this could be potentially partly explained with QS‐mediated competitive interactions with other bacteria (Harrison, Paul, Massey, & Buckling, 2008). There are several mutually nonexclusive explanations for reduced *P. aeruginosa* growth in the presence of competitors. First, competition for limited resources was likely stronger in the presence of other bacterial species leading to lower *P. aeruginosa* densities in polymicrobial pathogen communities. Second, interference competition could have directly reduced *P. aeruginosa* growth directly. For example, *S. maltophilia* has been observed to influence *P. aeruginosa* biofilm architecture and protein synthesis (Ryan et al. 2008), while *P. aeruginosa* has been shown to have negative effects on *S. aureus* due to upregulation of antistaphylococcal substances such as pyocyanin and phenazine (Michelsen et al., 2014). Even though *S. aureus* has not been shown to have direct negative effects on *P. aeruginosa*, the *S. aureus* presence has been shown to favour the increase in the frequency of QS‐deficient mutants (Harrison et al., 2008). In line with this study, it has been found that a QS‐positive PAO1 strain interacts more negatively with *S. aureus* compared to a QS‐negative *lasR* strain (Michelsen et al., 2014). Recent evidence suggests that reduced antagonism between *S. aureus* on *P. aeruginosa lasR* mutants could be due to metabolic divergence (Frydenlund Michelsen et al., 2015). However, more detailed community‐level experiments are needed to understand these dynamics more profoundly.

The negative effect of phage was clearest in PAO1 monoculture and generally in the presence of *S. aureus* with both pathogen genotypes. The presence of *S. maltophilia* did not affect phage efficiency with the PAO1 strain and even increased the *lasR* densities in the presence of phage (Figure 1a‐b), while phage had no effects on *P. aeruginosa* densities in the presence of both *S. maltophilia* and *S. aureus*. Together these results suggest that phages can reduce *P. aeruginosa* densities additively in the presence of competitors but that this effect depends on the strength of competition and the composition of the competing bacterial community. Interestingly, phage presence decreased and increased the total bacterial densities of polymicrobial *lasR* and PAO1 communities, respectively. Reduction in PAO1 frequency by the phage could have led to a competitive release and increased the growth of *S. aureus, S. maltophilia* and total bacterial densities. Conversely, resource competition was likely more intense and more symmetric within *lasR* communities due to stronger levels of phage resistance evolution (and hence higher *P. aeruginosa* density). Lastly, it has been shown that phage selection can impose relatively higher competitive cost for the PAO1 compared to the *lasR* strain due to upregulation of siderophore production (Vasse, Torres‐Barcelo, & Hochberg, 2015). Such metabolic cost could also potentially explain relatively poorer PAO1 growth in the presence of competitors even in the nonsocial culture conditions used in this experiment. In addition to demographic explanations, the potential changes at the gene expression level warrant thus further investigation in the future.

In line with the bacterial density data, the phage abundances were also generally lower in the presence of competitors and this effect was clearer with the PAO1 strain that suffered more heavily from competition compared to the *lasR* strain. Competition‐mediated reduction in bacterial and phage densities correlated with reduced levels of resistance evolution, and as a result, PAO1 strain evolved lower levels of resistance compared to the *lasR* strain. Simple demographic effects that weaken the strength of selection via reduced bacteria and phage encounter rates and lowered mutation supply rate (Lopez‐Pascua & Buckling, 2008) could thus be important for the evolutionary outcomes of phage therapy in polymicrobial infections. We also found that phages coevolved to be more infective during the selection experiment as demonstrated by higher levels of resistance of evolved bacteria to the ancestral compared to evolved phage populations. In line with the population dynamics data, the coevolutionary signal was stronger in *lasR* pathogen communities where both bacterial and phage densities were also higher. Bacterial competition did not thus constrain only the bacterial resistance but also the phage infectivity evolution and the trajectory of phage–bacteria coevolution.

Also, some underlying genetic differences could have affected PAO1 and *lasR* strain response to phages. It has been shown that removing, altering and concealing cell surface receptors can prevent phage adsorption (Seed, 2015) and that a functional QS system is important for regulating such phage defences (Hoyland‐Kroghsbo et al., 2013; Taj et al., 2014; Tan et al., 2015). In contrast to these findings, we found that QS‐defective strains were able to evolve higher levels of resistance to phages especially in the presence of bacterial competitors. A similar pattern has been found before, where the loss of QS impaired bacterial twitching motility leading to elevated resistance to pili‐specific phages (Glessner, Smith, Iglewski, & Robinson, 1999). Even though the PT7 target receptor is unknown, both the PAO1 and *lasR* strains were equally susceptible to the phage in the beginning of the experiment. This suggests that initial differences in PAO1 and *lasR* strains’ QS ability unlikely drove the long‐term differences in the bacterial resistance and phage infectivity evolution. Phage receptors are also often important for other purposes including nutrient uptake (Lenski & Levin, 1985), and hence, mutations in phage receptors often reduce bacterial competitive ability. In support for this, we found that both evolved PAO1 and *lasR* monoculture strains suffered reduced growth in the absence of phages and competitors if they had evolved in the presence of a phage during the selection experiment. Interestingly, while competitors increased the magnitude of the growth cost with PAO1 strain, competitors had no effect or even a positive effect on *lasR* growth. One explanation for this is that less antagonistic interactions between the *lasR* and competitors allowed more rapid accumulation of compensatory mutations during the selection experiment due to relatively large population size and mutation supply rate compared to PAO1 strain. We also found that evolved PAO1 strain grew better in the growth media compared to *lasR* strain when the bacteria had evolved in the absence of a phage and competitors. This suggest that functional QS system could help *P. aeruginosa* to adapt to abiotic environmental conditions potentially due to depressing of growth‐limiting intracellular metabolism (Asfahl, Walsh, Gilbert, & Schuster, 2015). In the community context, our results suggest that even though both focal pathogen genotypes were able to evolve resistance to phage, the PAO1 strain suffered more severe costs of adaptation due to both competition and phage.

Our results have important implications for the development of phage therapies in the context of polymicrobial infections. First, selection for phage resistance could be weaker in polymicrobial communities due to a competition‐mediated reduction in the focal pathogen density and relatively higher pleiotropic costs of adaptation. Competition could thus enhance the phage efficacy when treating acute CF and burn infections that are commonly co‐infected by QS signalling *P. aeruginosa*,* S. aureus* and *S. maltophilia* (Harrison, 2007; Turner et al., 2014). However, in contrary, *P. aeruginosa* resistance evolution to phages could be a more severe problem in chronic polymicrobial CF infections that are often dominated by *P. aeruginosa* mutants that have lost QS signalling ability during the long‐term adaptation (Andersen et al., 2015; Marvig et al., 2014; Smith et al., 2006). Interestingly, we found that higher levels of *lasR* strain resistance evolution were correlated with the higher rate of phage infectivity evolution, which could open up avenues for pre‐adapting phages to be more infective before clinical phage therapy treatments (Betts et al., 2013; Friman et al., 2016). Moreover, it would be interesting to investigate whether our results hold when multiple phage species are applied as a phage cocktail. We also want note that it is possible that both *S. aureus* and *S. maltophilia* strains evolved during the selection experiment. For example, it is known that *P. aeruginosa* can promote the formation of small colony variants with *S. aureus* leading to changes in virulence and antibiotic resistance (Frydenlund Michelsen et al., 2015; Hoffman et al., 2006). It is thus important to expand the study the evolutionary effects of competition and phage selection across the whole polymicrobial community in the future and also link these phenotypic changes with the changes at the genotypic level.

In conclusion, here, we show that the presence of competitors can modulate the phage‐mediated effects on a focal pathogen. Crucially, phage selection imposed weaker selection for resistance evolution when the effect of competition with the focal pathogen was strong. Moreover, while the phage presence indirectly reduced the total bacterial loads in weakly virulent *lasR* pathogen communities, phages increased the total bacterial densities in highly virulent PAO1 pathogen communities. Bacterial competition is thus likely to be an important factor affecting both the ecological and evolutionary outcomes of phage therapy in polymicrobial infections. From a therapeutic perspective, the fact that overwhelming phage numbers were not able to eradicate *Pseudomonas* even in the presence of competitors reinforces the importance of studying phage–bacteria interactions in the polymicrobial context.

## Data Archiving Statement

Data available from the Dryad Digital Repository: http://dx.doi.org/10.5061/dryad.8v234
