# Supplementary material for: Bacterial competition and quorum‐sensing signalling shape the eco‐evolutionary outcomes of model in vitro phage therapy
Source: Evol Appl. 2016 Dec 20;10(2):161–9. doi: 10.1111/eva.12435 (PMC5253424; doi:10.1111/eva.12435)
Supplement: Supplementary file 1 [file EVA-10-161-s001.docx]

**SUPPLEMENTARY INFORMATION**

**Supplementary table 1: Statistical models and F-statistics for *P. aeruginosa* bacterial densities**

Linear model predicting *P. aeruginosa* density as a function of phage treatment (present or absent), *P. aeruginosa* genotype (QS signalling or non-signalling) and competition (presence of *S. aureus*, *S. maltophilia* or both *S. aureus* and *S. maltophilia*).

| **Model Terms** | **Degrees of Freedom** | **Degrees of Freedom Error** | **F-Value** | **P-Value** |
| --- | --- | --- | --- | --- |
| **Phage treatment** | 1 | 64 | 8.67 | 0.005 |
| **Genotype** | 1 | 64 | 10.27 | 0.002 |
| **Competition** | 3 | 64 | 48.80 | >0.001 |
| **Phage treatment × Genotype** | 1 | 64 | 3.82 | 0.055 |
| **Phage treatment × Competition** | 3 | 64 | 7.70 | >0.001 |
| **Genotype × Competition** | 3 | 64 | 5.02 | 0.003 |
| **Phage treatment × Genotype × Competition** | 3 | 64 | 2.38 | 0.078 |

**Supplementary table 2: Statistical models and F-statistics for total bacterial densities in polymicrobial communities**

Linear model predicting total bacterial densities as a function of phage presence and *P. aeruginosa* genotype in polymicrobial communities.

| **Model Terms** | **Degrees of Freedom** | **Degrees of Freedom Error** | **F-Value** | **P-Value** |
| --- | --- | --- | --- | --- |
| **Phage** | 1 | 56 | 0.62 | 0.432 |
| **Genotype** | 1 | 56 | 2.10 | 0.152 |
| **Phage × Genotype** | 1 | 56 | 8.04 | 0.006 |

**Supplementary table 3: Statistical models and F-statistics for phage densities**

Mixed effects model predicting phage densities as a function of time, *P. aeruginosa* genotype (QS signalling or non-signalling) and competition (presence of *S. aureus*, *S. maltophilia* or both *S. aureus* and *S. maltophilia*).

| **Model Terms** | **Degrees of Freedom** | **Degrees of Freedom Error** | **F-Value** | **P-Value** |
| --- | --- | --- | --- | --- |
| **Time** | 3 | 30.35 | 17.34 | <0.001 |
| **Genotype** | 1 | 32.10 | 1.74 | 0.195 |
| **Competition** | 3 | 32.10 | 2.28 | 0.097 |
| **Genotype × Competition** | 3 | 32.10 | 2.96 | 0.047 |
| **Time × Genotype** | 3 | 32.35 | 2.63 | 0.067 |
| **Time × Competition** | 9 | 30.37 | 1.07 | 0.411 |
| **Time × Competition × Genotype** | 9 | 30.37 | 1.82 | 0.109 |

**Supplementary table 4: Statistical models and F-statistics for evolution of *P. aeruginosa* phage resistance**

Linear model predicting *P. aeruginosa* resistance to phage as a function of phage origin (ancestral or contemporary phage from own population), *P. aeruginosa* genotype (QS signalling or non-signalling) and competition (presence of *S. aureus*, *S. maltophilia* or both *S. aureus* and *S. maltophilia*).

| **Model Terms** | **Degrees of Freedom** | **Degrees of Freedom Error** | **F-Value** | **P-Value** |
| --- | --- | --- | --- | --- |
| **Phage origin** | 1 | 62 | 25.38 | <0.001 |
| **Genotype** | 1 | 62 | 35.94 | <0.001 |
| **Competition** | 3 | 62 | 2.14 | 0.109 |
| **Phage Origin × Genotype** | 1 | 62 | 4.15 | 0.046 |
| **Phage Origin × Competition** | 3 | 62 | 6.94 | <0.001 |
| **Phage Origin × Genotype × Competition** | 3 | 62 | 1.05 | 0.374 |

**Supplementary table 5: Statistical models and F-statistics for evolution of *P. aeruginosa* pleiotropic growth cost due to competition and phages**

Linear model predicting the growth of evolved *P. aeruginosa* bacteria in the absence of phage as a function of phage treatment during the selection experiment (present or absent), *P. aeruginosa* genotype (QS signalling or non-signalling) and the competition during the selection experiment (presence of *S. aureus*, *S. maltophilia* or both *S. aureus* and *S. maltophilia*).

| **Model Terms** | **Degrees of Freedom** | **Degrees of Freedom Error** | **F-Value** | **P-Value** |
| --- | --- | --- | --- | --- |
| **Phage** | 1 | 63 | 10.00 | 0.002 |
| **Genotype** | 1 | 63 | 0.05 | 0.822 |
| **Competition** | 3 | 63 | 1.33 | 0.274 |
| **Phage × Genotype** | 1 | 63 | 8.53 | 0.005 |
| **Phage × Competition** | 3 | 63 | 1.59 | 0.199 |
| **Genotype × Competition** | 3 | 63 | 2.38 | 0.078 |
| **Phage × Genotype × Competition** | 3 | 63 | 0.07 | 0.977 |

Same model as above but competition set as a two level factor (competition vs. no competition).

| **Model Terms** | **Degrees of Freedom** | **Degrees of Freedom Error** | **F-Value** | **P-Value** |
| --- | --- | --- | --- | --- |
| **Phage** | 1 | 71 | 13.36 | <0.001 |
| **Genotype** | 1 | 71 | 2.34 | 0.131 |
| **Competition** | 1 | 71 | 1.88 | 0.175 |
| **Phage × Genotype** | 1 | 71 | 6.27 | 0.015 |
| **Phage × Competition** | 1 | 71 | 3.43 | 0.068 |
| **Genotype × Competition** | 1 | 71 | 7.08 | 0.010 |
| **Phage × Genotype × Competition** | 1 | 71 | 0.11 | 0.916 |

**Supplementary figure 1.** The comparison of total bacterial population densities in the end of the selection experiment between different treatments (CFU denotes for colony forming units per mL). All bars show ±1 s.e.m.
